# Early-Life Exposure to Ambient Air Quality and Infant Health-Related Quality of Life: A Longitudinal Multi-Center Cohort in China

**DOI:** 10.3390/toxics14050371

**Published:** 2026-04-26

**Authors:** Yulin Wu, Ju Chen, Siting Zheng, Jieling Luo, Zhiyong Xie, Yi Liu, Mingxian Wu, Suxia Sun, Zheqing Zhang

**Affiliations:** Department of Nutrition and Food Hygiene, Guangdong Provincial Key Laboratory of Tropical Disease Research, School of Public Health, Southern Medical University, Guangzhou 510515, China; yl15625502401@163.com (Y.W.); cj1668339492@smu.edu.cn (J.C.); zhengst0302@smu.edu.cn (S.Z.); luojl22320185@smu.edu.cn (J.L.); xzy22320198@smu.edu.cn (Z.X.); liuyi22420161@smu.edu.cn (Y.L.); mingxianwu@smu.edu.cn (M.W.)

**Keywords:** air quality, infant health, health-related quality of life, mixed-effects model, China

## Abstract

Air pollution poses a major public health threat, yet longitudinal evidence on its impact on infant health-related quality of life (HRQoL) remains limited. This study investigated the longitudinal associations between early-life exposure to outdoor air pollution and infant HRQoL, focusing on psychological and physiological domains. Between November 2021 and September 2022, 779 mother–newborn pairs were recruited, with 696 completing follow-up at 12 months. HRQoL was assessed at 1, 4, 6, and 12 months using the Pediatric Quality of Life Inventory™, and exposures to PM_2.5_, PM_10_, NO_2_, and SO_2_ were analyzed using linear mixed-effects models adjusted for infant sex, household income, sibling status, and other covariates. Higher concentrations of all pollutants were associated with lower total HRQoL scores. Stratified analyses showed that PM_2.5_, PM_10_, and SO_2_, but not NO_2_, were associated with steeper age-related declines in total scores. Inverse associations with psychosocial health were consistent across pollutants, with pronounced age-related declines in high-exposure groups; for physical health, only SO_2_ and NO_2_ remained significant after adjustment, with modest age-related changes. These findings suggest that early-life air pollution exposure is associated with smaller gains in HRQoL during infancy, particularly in psychosocial well-being, highlighting the importance of improving air quality to support early development.

## 1. Introduction

Clean air is a fundamental requirement for the maintenance of optimal health and well-being [1]. However, the phenomenon of rapid urbanization has significantly contributed to air pollution, thereby creating an urgent environmental threat to human health [2]. As urban areas expand and their populations grow, the concomitant increase in industrial activities, vehicular traffic, and construction can result in a deterioration of air quality [3]. Even when pollution levels remain below the thresholds set by relevant air quality guidelines, the effects of air pollution can still be evident [1,4]. Infants and young children face an increased risk due to their heightened biological vulnerability and the unique exposure patterns to air pollution that they experience [5,6]. Understanding the impact of air pollution on infant health is crucial for developing effective interventions and policies to protect this vulnerable population.

A substantial body of epidemiological research in pediatric populations indicates that exposure to air pollution not only exacerbates respiratory diseases but may also increase the risk of preterm birth, low birth weight, neurodevelopmental disorders, and pediatric cancers [7]. These adverse effects are further supported by mechanistic toxicological evidence demonstrating that air pollutants can trigger oxidative stress, neuroinflammation, and blood–brain barrier disruption, thereby affecting neurodevelopment and physical health [8,9,10]. Quality of life (QoL) is typically conceptualized as the quality of an individual’s daily life, encompassing an appraisal of their well-being or otherwise [11]. Health-related quality of life (HRQoL) focuses on aspects of well-being influenced by health status. It considers not only the infant‘s physical health but also their emotional and developmental needs, thereby providing a more comprehensive understanding of overall well-being [12]. The PedsQL^TM^ has been widely used to assess HRQoL in children with various health conditions, including asthma, cancer, and diabetes [13]. While self-report versions exist for older children, proxy versions have been developed for younger children and infants. The PedsQL^TM^ Infant Scales were specifically validated for children aged 1–24 months using parent proxy report, and subsequent studies have successfully used parent-reported PedsQL in infants with suspected genetic conditions [14]. Thus, despite the inherent limitation of proxy reporting, the PedsQL^TM^ Infant Scales provide a well-established instrument for assessing HRQoL in this age group. However, there is currently no research examining the effects of air pollution on infants’ QoL. Single-center studies are conducted at one site, so their findings may reflect local air pollution mixtures (e.g., PM_2.5_ composition varies between northern and southern China) and local population characteristics. These factors limit generalizability. Therefore, we designed a multi-center study across several Chinese cities to test whether the association between early-life air pollution exposure and infant HRQoL is consistent across different environmental settings.

The objective of this paper is to further validate the aforementioned relationship and to explore the underlying mechanisms by means of a comprehensive analysis of air pollution and infant HRQoL scores in different cities in China. This will provide a crucial scientific foundation for the development of more effective public health policies aimed at safeguarding infant health and development.

## 2. Materials and Methods

### 2.1. Study Population

The study utilized data from an ongoing prospective birth cohort study conducted in six Chinese cities (Nanjing, Chengdu, Xi’an, Wuhan, Guangzhou and Qingdao respectively) [15]. At baseline (2021–2023), we recruited pregnant women at 37 weeks of gestation or more and conducted face-to-face interviews at the hospitals partnering with each study central site. Women were eligible if they met the following criteria: (1) healthy mothers with breastfeeding intentions; (2) 18 years of age and older; and (3) voluntarily provided written informed consent. Mother–infant pairs were invited to a series of clinical visits or interviews at 1 month, 4 months, 6 months, and 1 year postpartum. This study included a total of 769 infants who completed the one-year follow-up period (Figure S1). The study protocol was approved by the ethics committees of all participating centers. Written informed consent was obtained from all participants before inclusion.

### 2.2. Outcome Measurements

The HRQoL score of infants was evaluated using the Infant Quality of Life Scale (PedsQL^TM^, Mapi Research Trust, Lyon, France) [13]. Parents of infants aged between 1 and 12 months were asked to respond to 36 questions, which were grouped into five dimensions: physical functioning, physical symptoms, emotional functioning, social functioning and cognitive functioning. A higher score indicates a superior level of HRQoL. A five-point Likert scale, ranging from 0 (never) to 4 (almost always), was employed to assess the degree to which each question was answered in the affirmative. The questions were not weighted. The scores were subsequently transformed onto a scale ranging from 0 to 100. Items are reverse scored and linearly transformed to a 0~100 scale (0 = 100, 1 = 75, 2 = 50, 3 = 25, 4 = 0), so that higher scores indicate better HRQoL. The Total Scale Score is computed as the sum of all the items on the PedsQL^TM^ Infant Scales divided by the number of items answered. Scale scores are computed as the sum of the items divided by the number of items answered (this accounts for missing data). The Physical Health Summary Score is computed as the sum of the items over the number of items answered in the Physical Functioning and Physical Symptoms Scales. The Psychosocial Health Summary Score is computed as the sum of the items over the number of items answered in the Emotional, Social, and Cognitive Functioning Scales. If more than 50% of the items in the scale are missing, the Scale score is not computed [16]. All questionnaires were administered through face-to-face interviews by trained researchers. Missing data were minimal: only three questionnaires had 1–4 missing items each. These missing items were imputed using the mean of the corresponding dimension. Since no scale exceeded the 50% missing threshold, all scale scores were successfully computed. The internal consistency of the PedsQL^TM^ Infant Scales in our sample was excellent. Across the four time points (1, 4, 6, and 12 months), Cronbach’s α ranged from 0.906 to 0.930 for the total HRQoL score, from 0.823 to 0.860 for the physical health dimension, and from 0.877 to 0.910 for the psychosocial health dimension. These values exceed the commonly accepted threshold of 0.70, indicating that parent-proxy reports were highly reliable in this cohort.

### 2.3. Environmental Data

Daily average concentrations of PM_2.5_, PM_10_, NO_2_, and SO_2_ were obtained from the National Urban Air Quality Real-time Release Platform maintained by the China National Environmental Monitoring Centre (CNEMC), covering all six cities under study. Monitoring stations were initially selected from all available sites established within each study city (total *n* = 83). In China, healthcare-seeking behavior is typically localized; mothers and infants attend nearby maternal and child health hospitals for routine care and study recruitment. Thus, participants naturally resided within a reasonable distance from the recruitment centers. Consequently, we restricted inclusion to monitoring stations located within a 20 km radius of these centers, resulting in the inclusion of 55 stations. These 55 fixed-site monitoring stations provided continuous measurements of outdoor air pollution throughout the study period (2021–2023).

Given the study’s focus on infant HRQoL assessed at four postpartum intervals (1, 4, 6, and 12 months), we calculated average exposure concentrations for four developmental windows: birth to 1 month, 1 to 4 months, 4 to 6 months, and 6 to 12 months. For each pollutant, a daily area-weighted mean concentration was derived from stations within the 20 km radius of each participant’s recruitment center. Minor data gaps (<5% of daily records) were imputed using a 3-day moving average centered on the missing day.

These windows were selected based on established infant developmental milestones. Huang et al. identified 1, 4, and 6 months as key turning points in sleep/wake development: nighttime sleep consolidates markedly during 1–4 months (longest continuous sleep reaching 6.8 h by 4 months), and after 6 months night awakenings decrease to <0.5 per night [17]. Choi et al. demonstrated that exclusive breastfeeding during the first 4 months was associated with better communication and cognitive development at 6 and 12 months, validating 4, 6, and 12 months as critical follow-up time points [18]. Thus, these windows reflect meaningful physiological and behavioral transitions.

### 2.4. Covariate Assessments

At the baseline interview, we collected sociodemographic characteristics and lifestyle factors of mothers and their partners, including the highest level of education attained by both parties, annual household income (a well-established correlate of infant development [19]), maternal smoking exposure (yes/no), sex of the infant (male or female), presence of siblings (yes/no, which may influence infant mental health and QoL [20]), mode of delivery, and gestational age. Infants’ feeding patterns (exclusive breastfeeding, formula feeding, or mixed feeding) were recorded at each visit at ages 1, 4, 6, and 12 months. Additionally, the season (spring, summer, autumn, winter) at the time of each follow-up visit was documented as a time-varying covariate. Infant age (in months) was treated as a continuous variable in the linear mixed-effects models to estimate age-related trajectories, as age is a critical time-varying factor for growth and development requiring precise control.

### 2.5. Statistical Analysis

The demographic results are presented as mean ± SD or *n* (%). For a description of the concentrations of air pollution, QoL scores, the physical health summary score and the psychosocial health summary score, the results are presented as median and 25th–75th percentile (Q1 and Q3).

Linear mixed-effects models (LMMs) were employed to examine longitudinal associations between air pollution (PM_2.5_, PM_10_, SO_2_, and NO_2_) as continuous variables and three key infant health outcomes: overall QoL, composite physical health score, and composite psychosocial health score during the first year of life. Three separate models were constructed, one for each outcome variable. Fixed effects incorporated into each model included the specific pollutant concentration, infant age (in months), and their interaction term. Covariates comprised infant sex, annual household income, presence of siblings, mode of delivery, gestational age, feeding patterns, maternal smoking exposure, and season, as well as their interactions with infant age (months). All models included a random intercept to account for within-subject variability. Infant age (in months) was treated as a continuous variable in the LMMs to estimate age-related trajectories. For descriptive purposes and figure presentation, age was grouped into four discrete time points (1, 4, 6, and 12 months).

Each pollutant’s concentration was further categorized into tertiles (low, medium, high) based on its distribution within each of the four follow-up periods (birth to 1 month, 1 to 4 months, 4 to 6 months, 6 to 12 months). To further investigate differences in the association slopes across air pollution concentration levels, pairwise comparisons of estimated marginal means (EMMs) between concentration tertiles (high vs. medium, high vs. low, medium vs. low) were conducted. The False Discovery Rate (FDR) method was applied to correct for multiple testing. A two-sided *p*-value ≤ 0.05 was considered statistically significant. All linear mixed-effects models were fitted using the lme4 package in R (version 4.3.3; R Foundation for Statistical Computing, Vienna, Austria). Data analysis and visualization were performed within the R environment.

## 3. Results

### 3.1. Participant Characteristics

A total of 779 mother–infant dyads were enrolled in the cohort between 19 November 2021 and 23 September 2023. Baseline characteristics of the 769 dyads included in the analysis are presented in Table 1. Most parents attained higher education: 69.96% of mothers (*n* = 767; 2 missing) and 69.83% of fathers held a college diploma or associate degree, while 15.73% of both parents had a postgraduate degree or higher.

Table 2 presents the concentrations of air pollution across different infant age intervals. The median concentration of PM_2.5_ increased from 27.13 μg/m^3^ at 0–1 month to 36.84 μg/m^3^ at 6–12 months. Similarly, PM_10_ concentrations rose from 51.93 μg/m^3^ at 0–1 month to 70.89 μg/m^3^ at 6–12 months (IQR: 28.43). NO_2_ concentrations showed a monotonic increase over time, from 29.93 μg/m^3^ at 0–1 month to 33.74 μg/m^3^ at 6–12 months (IQR: 8.54). In contrast, SO_2_ concentrations remained relatively stable across infancy, with median values ranging from 5.81 μg/m^3^ at 6 months to 6.17 μg/m^3^ at 12 months (IQR: 2.15–4.18).

### 3.2. Associations of Air Pollution with Total HRQoL

Table 3 presents the interaction effects between continuous air pollutant concentra-tions and infant age on HRQoL scores. In crude models, all pollutants showed significant negative interactions for total, physical, and psychosocial HRQoL (all *p* < 0.05). After adjustment, significant negative interactions with age were observed for total HRQoL for PM_2.5_ (*β* = −0.012, *p* < 0.001), PM_10_ (*β* = −0.007, *p* < 0.001), and SO_2_ (*β* = −0.079, *p* < 0.001), but not for NO_2_ (*β* = −0.009, *p* = 0.084). For physical health, only SO_2_ remained significant after adjustment (*β* = −0.054, *p* < 0.001). For psychosocial health, all four pollutants demon-strated strong negative interactions after adjustment (adjusted *β* range: −0.107 to −0.012, all *p* < 0.05). These results suggest that the detrimental impact of air pollution on infant HRQoL, particularly on psychosocial well-being, becomes more pronounced with in-creasing age.

**Figure 1:**
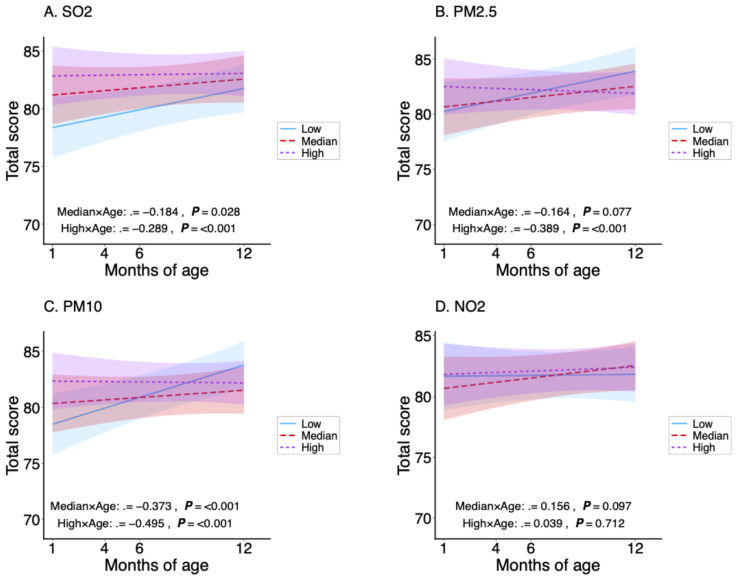
Age-related changes in total infant health-related quality of life (HRQoL) scores stratified by low, median, and high exposure levels of PM_2.5_, PM_10_, NO_2_, and SO_2_. Blue, red, and purple lines represent low, median, and high exposure categories, respectively. Lines represent fitted estimates from linear mixed-effects models adjusted for infant sex, household income, sibling status, mode of delivery, gestational age, feeding patterns, maternal smoking exposure, season, and their interactions with age (months). Shaded areas indicate 95% confidence intervals.

**Figure 2:**
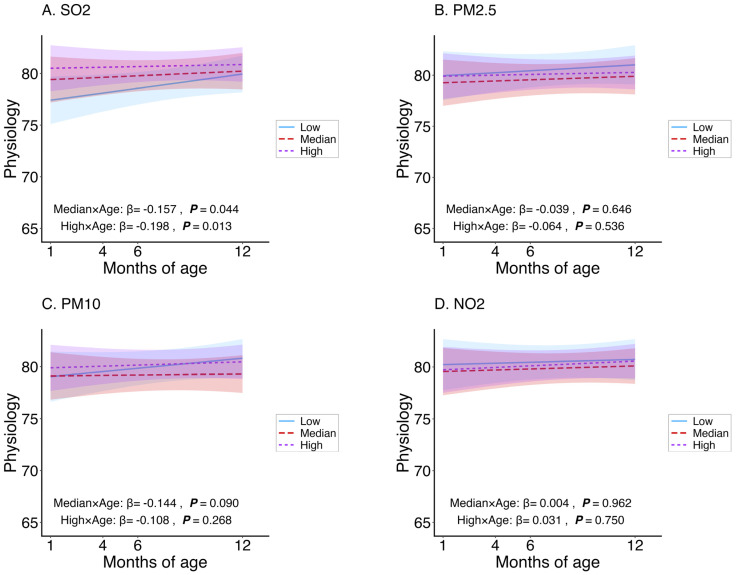
Age-related changes in infant physical health composite scores stratified by exposure levels of PM_2.5_, PM_10_, NO_2_, and SO_2_, estimated using adjusted linear mixed-effects models. Blue, red, and purple lines represent low, median, and high exposure categories, respectively. Lines represent fitted estimates from linear mixed-effects models adjusted for infant sex, household income, sibling status, mode of delivery, gestational age, feeding patterns, maternal smoking exposure, season, and their interactions with age (months). Shaded areas indicate 95% confidence intervals.

**Figure 3:**
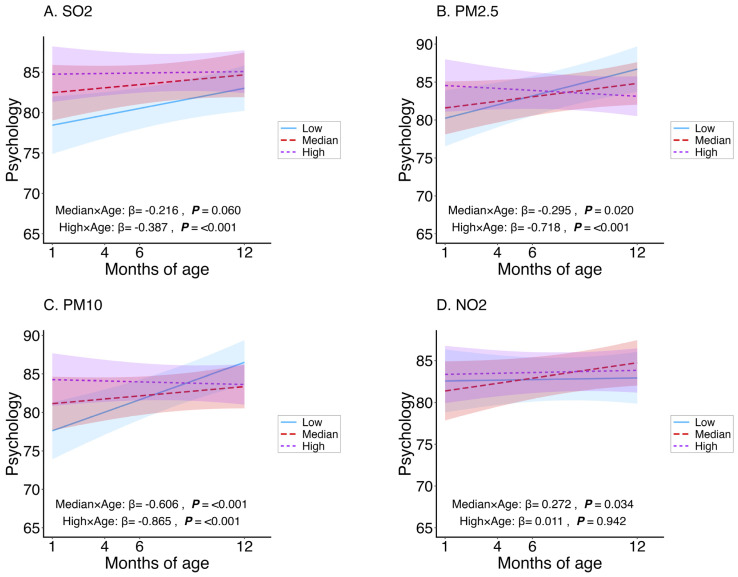
Age-related trajectories of infant psychosocial health composite scores according to low, median, and high exposure levels of PM_2.5_, PM_10_, NO_2_, and SO_2_. Blue, red, and purple lines represent low, median, and high exposure categories, respectively. Lines represent fitted estimates from linear mixed-effects models adjusted for infant sex, household income, sibling status, mode of delivery, gestational age, feeding patterns, maternal smoking exposure, season, and their interactions with age (months). Shaded areas indicate 95% confidence intervals.

The interaction term (exposure group × age) quantifies the age-related change in HRQoL across exposure levels; negative coefficients indicate steeper declines with age compared to the low-exposure group. For total HRQoL (Figure 1), the high-exposure groups showed significant negative interactions for PM_2.5_ (*β* = −0.389), PM_10_ (*β* = −0.495), and SO_2_ (*β* = −0.289), all *p* < 0.001. The median-exposure groups were significant for PM_10_ (*β* = −0.373, *p* < 0.001) and SO_2_ (*β* = −0.184, *p* = 0.028). NO_2_ had no significant interactions. For physical health (Figure 2), only SO_2_ was significant in both median (*β* = −0.157, *p* = 0.044) and high (*β* = −0.198, *p* = 0.013)-exposure groups. For psychosocial health (Figure 3), high-exposure groups exhibited strong negative interactions for PM_2.5_ (*β* = −0.718), PM_10_ (*β* = −0.865), and SO_2_ (*β* = −0.387), all *p* < 0.001. Median groups were significant for PM_2.5_ (*β* = −0.295, *p* = 0.020) and PM_10_ (*β* = −0.606, *p* < 0.001). NO_2_ showed a positive interaction in the median group (*β* = 0.272, *p* = 0.034) but no effect in the high group. Overall, adverse age-related associations were strongest and most consistent for psychosocial health, particularly for particulate matter.

## 4. Discussion

This cohort study systematically evaluated the dynamic effects of outdoor air pollution on infant HRQoL, including physical (physiological functioning, physical symptoms) and psychological (emotional, social, cognitive functioning) domains. We found that higher concentrations of SO_2_, PM_2.5_, PM_10_, and NO_2_ were consistently associated with lower total QoL scores, with particularly strong and stable associations observed for psychosocial health. Age-related analyses suggested steeper declines in psychosocial outcomes at higher exposure levels, especially for particulate matter and SO_2_, whereas associations with physical health were comparatively modest. These findings indicate that infant psychosocial well-being may be especially sensitive to air pollution exposure during early development.

Linear mixed-effects models demonstrated significant positive associations between exposure to SO_2_, PM_2.5_, and PM_10_ and the total QoL score as well as psychosocial functioning at baseline. However, the interaction analysis showed that higher concentrations of pollutants such as PM_2.5_, PM_10_, SO_2_, and NO_2_ were associated with slower growth rates in QoL scores compared to lower concentrations. Specifically, higher concentrations of PM_2.5_, PM_10_, SO_2_, and NO_2_ were associated with slower growth rates in psychosocial domain scores, while higher SO_2_ exposure levels were associated with slower growth rates in physical domain scores. Although studies on air pollution and QoL in infants are lacking, related evidence is available in adult populations. A comprehensive survey of the Korean population using data from the 2013 Korea Community Health Survey, which employed the EuroQol 5-Dimension (EQ-5D) health questionnaire to assess QoL, showed a correlation between increased annual concentrations of PM_10_ and NO_2_ and decreased QoL [21]. Additionally, available evidence suggests that air pollution may be adversely associated with psychological and neurodevelopmental trajectories in infants, as well as increasing the risk of physical diseases such as respiratory infections [22]. Air pollution is increasingly recognized for its adverse effects on psychological and neurodevelopmental trajectories. A compelling and expanding body of research, including work from Chinese cohorts, reports associations between early-life exposure to PM_2.5_ and NO_2_ and poorer neurodevelopment or increased behavioral problems in young children [23,24]. This is supported by international studies linking similar exposures to long-term neurodevelopmental consequences, including impaired cognitive skills associated with early-life PM_2.5_ exposure in a Spanish cohort and increased risks of behavioral problems linked to prenatal pollution in a Japanese population [25,26]. Our study contributes to this evidence by quantifying how air pollution exposure may attenuate the normative, age-related improvements in infant QoL, thereby providing a novel measure of its impact on early well-being.

Compared with current air quality guidelines, median PM_2.5_ (25.2–36.8 μg/m^3^) and PM_10_ (48.9–70.9 μg/m^3^) exceeded both WHO 2021 annual guidelines (5 and 15 μg/m^3^) [1] and Chinese Grade II standards (25 and 50 μg/m^3^) [27]. NO_2_ medians (28.4–33.7 μg/m^3^) exceeded the WHO annual guideline of 10 μg/m^3^ [1] and were slightly above the Chinese Grade I standard of 30 μg/m^3^ [27], while SO_2_ (approximately 6 μg/m^3^) was well below both limits. In our adjusted models, each unit increase in pollutant concentration was associated with a reduction in total HRQoL ranging from 0.007 to 0.079 points. More importantly, infants in the high-exposure groups exhibited steeper age-related declines in total HRQoL, reaching up to −0.495 for PM_10_, as well as a decline of −0.865 in psychosocial health compared with those in the low-exposure group. Although the observed effect sizes were modest, they were statistically robust and consistent across pollutants. Given that air pollution is a ubiquitous exposure affecting entire populations, even these modest associations may translate into meaningful public health burdens. Our findings suggest that reducing air pollution during early infancy could yield population-level benefits for infant HRQoL, particularly in the psychosocial domain, reinforcing the need for stricter air pollution control.

Previous animal studies have provided substantial evidence of the adverse effects of air pollution on the CNS [28]. Inhaled air pollution can translocate to the CNS through multiple pathways, including the olfactory epithelium, blood–brain barrier, and gastrointestinal tract [29]. In an experiment using diesel exhaust-origin secondary organic aerosol (DE-SOA) as an air pollution model, perinatal exposure to DE-SOA was shown to cause neuroimmune response dysregulation in rats [30]. Specifically, exposure to DE-SOA led to a significant reduction in vascular endothelial growth factor (VEGF) mRNA in the hippocampus of male rats, where VEGF plays a critical role in cerebrovascular formation, neurogenesis, and synaptic plasticity [31]. It enhances blood–brain barrier permeability, supports neuronal growth, prevents apoptosis, and regulates hippocampal function. Conversely, stress has been shown to reduce its expression, thereby impairing these processes [32]. Notably, the developing brain is highly vulnerable to toxic substances [33]. Neuroinflammation induced by air pollution exposure has the potential to alter innate immune responses and even influence the development of human neurodegenerative diseases [34]. Additionally, pollutants such as PM and NO_2_ have been demonstrated to irritate the respiratory tract, exacerbate asthma symptoms, and reduce lung function, particularly in infants with incompletely developed lungs. These particles can penetrate lung tissue, triggering oxidative stress and cellular damage, which in turn induce inflammation. Multiple pollutants (e.g., NO_2_, PM_2.5_) stimulate the release of pro-inflammatory cytokines (e.g., IL-6, TNF-α), which may lead to systemic inflammation, affect multi-organ function, and increase infant fatigue, poor physical function, and other discomfort symptoms [35,36]. However, further research is needed to deeply understand the specific components of outdoor air pollution that cause CNS damage and physical discomfort, as well as the human-relevant molecular mechanisms involved. While our study has notable strengths in exploring the relationship between outdoor air pollution and HRQoL scores, it is not without limitations. First, and most importantly, exposure assessment was based on outdoor fixed-site monitoring data, which introduces non-differential measurement error. This typically biases effect estimates toward the null, meaning our reported associations are likely conservative [37]. Specifically, exposure was assigned based on monitoring stations within a 20 km radius of recruitment centers. However, this spatial resolution may not capture fine-scale urban pollution gradients and does not account for individual-level variability in residential mobility, indoor and outdoor exposure, microenvironment, or time–activity patterns, thereby increasing the likelihood of exposure misclassification. Secondly, infants spend the majority of their time indoors, which introduces a random and undifferentiated exposure error that may result in an underestimation of the effects of outdoor air pollution. While data on major outdoor air pollution were comprehensively analyzed, influences such as indoor humidity or condensation and tobacco smoke were not taken into account [38]. Thirdly, HRQoL scores were obtained using parent-reported indicators via the PedsQL^TM^ questionnaire. While these data have been validated, they may be subject to potential biases, including recall bias or subjective interpretations of infant health and functioning. In addition, as recently highlighted by Powell et al., HRQoL assessment in early childhood is subject to several inherent limitations, including potential spillover effects from caregivers’ HRQoL, limited incorporation of subjectively experienced domains with validated behavioral correlates (e.g., crying as a proxy for pain or distress), and insufficient sensitivity to capture rapid developmental changes [39]. Future longitudinal studies incorporating more refined and developmentally sensitive measures of children’s physical and psychological health are warranted to validate our findings. Fourth, several relevant confounders were not assessed, including paternal mental health, breastfeeding quality, and paternal quality of life, which may have led to residual confounding.

## 5. Conclusions

This study found that early-life exposure to ambient air pollution was consistently associated with poorer infant HRQoL, particularly psychosocial health. Higher levels of SO_2_, PM_2.5_, PM_10_, and NO_2_ were associated with lower overall scores, with stronger and more stable effects on psychosocial outcomes that worsened with age, especially for particulate matter and SO_2_. In contrast, physical health showed smaller age-related effects. These findings suggest that reducing air pollution may help protect infant HRQoL during early development.

## Figures and Tables

**Table 1:** Demographic characteristics of 769 mother–child pairs.

Characteristics	*n* = 769
Maternal education	
Low ^1^	96 (12.48%)
Medium ^2^	538 (69.96%)
High ^3^	135 (17.56%)
Paternal education	
Low ^1^	111 (14.43%)
Medium ^2^	537 (69.83%)
High ^3^	121 (15.73%)
Annual household income (RMB)	
≤150,000	253 (32.99%)
150,000–300,000	385 (50.20%)
>300,000	129 (16.82%)
Gender	
Female	390 (50.72%)
Male	379 (49.28%)
Delivery	
Vaginal delivery	432 (56.18%)
Cesarean delivery	337 (43.82%)
Presence of siblings	
Yes	305 (39.66%)
No	464 (60.34%)
Total quality of life score	
At 1 month of age	76.97 [67.76; 88.82]
At 4 month of age	82.24 [75.16; 88.82]
At 6 month of age	83.55 [76.97; 91.45]
At 12 month of age	86.18 [78.29; 91.45]
Composite physical health score	
At 1 month of age	77.78 [69.44; 83.33]
At 4 month of age	80.56 [75.00; 86.11]
At 6 month of age	83.33 [76.39; 87.50]
At 12 month of age	83.33 [77.78; 87.50]
Composite psychosocial health score	
At 1 month of age	77.50 [63.75; 96.25]
At 4 month of age	83.75 [75.00; 93.75]
At 6 month of age	85.00 [77.50; 96.25]
At 12 month of age	87.50 [78.75; 96.25]

Note: ^1^ Low: high school and below. ^2^ Medium: college or university. ^3^ High: master’s degree and above.

**Table 2:** The concentrations of outdoor air pollutants to which subjects were exposed at each follow-up node.

Air Pollutants	Median [IQR]	Minimum	Maximum	IQR
SO_2_ (µg/m^3^)				
At 1 month of age	6.16 [5.19; 7.71]	3.55	10.23	2.52
At 4 month of age	6.07 [5.00; 7.15]	3.20	9.54	2.15
At 6 month of age	5.81 [5.03; 7.45]	2.72	11.78	2.42
At 12 month of age	6.17 [4.80; 8.98]	3.04	9.94	4.18
PM_2.5_ (µg/m^3^)				
At 1 month of age	27.13 [18.95; 40.55]	11.48	82.59	21.60
At 4 month of age	25.17 [19.69; 31.05]	13.84	85.16	11.36
At 6 month of age	26.53 [21.37; 37.13]	11.86	116.45	15.76
At 12 month of age	36.84 [26.45; 51.23]	17.19	95.19	24.78
PM_10_ (µg/m^3^)				
At 1 month of age	51.93 [36.38; 72.60]	23.99	182.19	36.22
At 4 month of age	48.92 [39.58; 59.92]	26.17	127.92	20.34
At 6 month of age	49.44 [41.22; 65.55]	24.69	174.10	24.33
At 12 month of age	70.89 [52.26; 80.69]	32.50	154.21	28.43
NO_2_ (µg/m^3^)				
At 1 month of age	29.93 [23.43; 41.40]	12.47	59.79	17.97
At 4 month of age	28.36 [24.11; 34.09]	16.03	50.27	9.98
At 6 month of age	30.19 [23.16; 36.72]	14.47	59.80	13.56
At 12 month of age	33.74 [29.84; 38.38]	19.02	53.28	8.54

Note: IQR, interquartile range. The daily average exposure concentration is the daily average exposure concentration at four time points: birth to 1 month of age, 1 month to 4 months of age, 4 months to 6 months of age, and 6 months to 12 months of age.

**Table 3:** The associations of air pollutants with infant quality of life scores.

Predictor	Total Infant Quality of Life Scores			Infant Physical Health Composite Scores			Infant Psychosocial Health Composite Scores		
	*β*	SE	*p*	*β*	SE	*p*	*β*	SE	*p*
SO_2_									
Model 1	−0.083	0.016	**<0.001**	−0.048	0.015	**0.002**	−0.120	0.022	**<0.001**
Model 2	−0.079	0.016	**<0.001**	−0.054	0.016	**0.001**	−0.107	0.023	**<0.001**
PM_2.5_									
Model 1	−0.014	0.002	**<0.001**	−0.004	0.002	**0.033**	−0.024	0.003	**<0.001**
Model 2	−0.012	0.003	**<0.001**	−0.002	0.003	0.390	−0.022	0.004	**<0.001**
PM_10_									
Model 1	−0.008	0.001	**<0.001**	−0.003	0.001	**0.015**	−0.013	0.002	**<0.001**
Model 2	−0.007	0.001	**<0.001**	−0.002	0.001	0.150	−0.012	0.002	**<0.001**
NO_2_									
Model 1	−0.022	0.004	**<0.001**	−0.012	0.004	**0.003**	−0.032	0.006	**<0.001**
Model 2	−0.009	0.005	0.084	−0.002	0.005	0.699	−0.017	0.007	**0.019**

Model 1: Crude model. Model 2: Adjusted for infant gender, family annual income, sibling status, delivery method, gestational week, feeding patterns, maternal smoking exposure, season, months of age, and pollutant concentration. The table only presents the interaction term coefficients (pollutant × months of age); non-standardized *β* values reflect the marginal effect of increasing pollutant concentration on quality of life score as age increases. Bold values indicate statistical significance (*p* < 0.05).

## Data Availability

The original contributions presented in this study are included in the article/Supplementary Materials. Further inquiries can be directed to the corresponding authors.

## Supplementary Materials

The following supporting information can be downloaded at: https://www.mdpi.com/article/10.3390/toxics14050371/s1, Figure S1: Screening and follow-up flow chart of study participants.

## References

[1] WHO (2021). WHO Global Air Quality Guidelines: Particulate Matter (PM_2.5_ and PM_10_), Ozone, Nitrogen Dioxide, Sulfur Dioxide and Carbon Monoxide.

[2] Piracha A., Chaudhary M.T. (2022). Urban Air Pollution, Urban Heat Island and Human Health: A Review of the Literature. Sustainability.

[3] Guo Y., Zhang Q., Lai K.K., Zhang Y., Wang S., Zhang W. (2020). The Impact of Urban Transportation Infrastructure on Air Quality. Sustainability.

[4] Myhre O., Låg M., Villanger G.D., Oftedal B., Øvrevik J., Holme J.A., Aase H., Paulsen R.E., Bal-Price A., Dirven H. (2018). Early life exposure to air pollution particulate matter (PM) as risk factor for attention deficit/hyperactivity disorder (ADHD): Need for novel strategies for mechanisms and causalities. Toxicol. Appl. Pharmacol..

[5] Lin Y.T., Shih H., Jung C.R., Wang C.M., Chang Y.C., Hsieh C.Y., Hwang B.F. (2021). Effect of exposure to fine particulate matter during pregnancy and infancy on paediatric allergic rhinitis. Thorax.

[6] Kwok M.K., Schooling C.M., Ho L.M., Leung S.S., Mak K.H., McGhee S.M., Lam T.H., Leung G.M. (2008). Early life second-hand smoke exposure and serious infectious morbidity during the first 8 years: Evidence from Hong Kong’s “Children of 1997” birth cohort. Tob. Control.

[7] Brumberg H.L., Karr C.J. (2021). Ambient Air Pollution: Health Hazards to Children. Pediatrics.

[8] Shang M., Tang M., Xue Y. (2023). Neurodevelopmental toxicity induced by airborne particulate matter. J. Appl. Toxicol..

[9] Liu F., Liu C., Liu Y., Wang J., Wang Y., Yan B. (2023). Neurotoxicity of the air-borne particles: From molecular events to human diseases. J. Hazard. Mater..

[10] Wylie A.C., Short S.J. (2023). Environmental Toxicants and the Developing Brain. Biol. Psychiatry.

[11] Jenney M.E., Kane R.L., Lurie N. (1995). Developing a measure of health outcomes in survivors of childhood cancer: A review of the issues. Med. Pediatr. Oncol..

[12] Irvine E.J. (1997). Quality of life issues in patients with inflammatory bowel disease. Am. J. Gastroenterol..

[13] Varni J.W., Limbers C.A., Neighbors K., Schulz K., Lieu J.E., Heffer R.W., Tuzinkiewicz K., Mangione-Smith R., Zimmerman J.J., Alonso E.M. (2011). The PedsQL^TM^ Infant Scales: Feasibility, internal consistency reliability, and validity in healthy and ill infants. Qual. Life Res..

[14] Smith H.S., Leo M., Goddard K., Muessig K., Angelo F., Knight S., Outram S., Kelly N.R., Rini C. (2024). Measuring health-related quality of life in children with suspected genetic conditions: Validation of the PedsQL proxy-report versions. Qual. Life Res..

[15] Wu J., Dong L., Sun Y., Zhao X., Gan J., Wang Z. (2024). The Effectiveness of Artificial Intelligence in Assisting Mothers with Assessing Infant Stool Consistency in a Breastfeeding Cohort Study in China. Nutrients.

[16] Fairclough D.L. (2010). Design and Analysis of Quality of Life Studies in Clinical Trials.

[17] Huang X.N., Wang H.S., Lu X.C., Jiang J.X., An L. (2009). [Development of sleep/wake patterns in infants during the first 12 months of life]. Zhonghua Er Ke Za Zhi.

[18] Choi H.J., Kang S.K., Chung M.R. (2018). The relationship between exclusive breastfeeding and infant development: A 6- and 12-month follow-up study. Early Hum. Dev..

[19] Li Z., Christensen G.M., Lah J.J., Marcus M., Russell A.G., Ebelt S., Waller L.A., Hüls A. (2022). Neighborhood characteristics as confounders and effect modifiers for the association between air pollution exposure and subjective cognitive functioning. Environ. Res..

[20] Silva W., Virtanen E., Kajantie E., Sebert S. (2024). Cognition, mental health and quality of life amongst siblings of preterm born children: A systematic review. Acta Paediatr..

[21] Shin J., Park J.Y., Choi J. (2018). Long-term exposure to ambient air pollutants and mental health status: A nationwide population-based cross-sectional study. PLoS ONE.

[22] Brauer M., Hoek G., Van Vliet P., Meliefste K., Fischer P.H., Wijga A., Koopman L.P., Neijens H.J., Gerritsen J., Kerkhof M. (2002). Air pollution from traffic and the development of respiratory infections and asthmatic and allergic symptoms in children. Am. J. Respir. Crit. Care Med..

[23] Li R., Jiang N., Liu Q., Huang J., Guo X., Liu F., Gao Z. (2017). Impact of Air Pollutants on Outpatient Visits for Acute Respiratory Outcomes. Int. J. Environ. Res. Public Health.

[24] Wang H., Zhang H., Li J., Liao J., Liu J., Hu C., Sun X., Zheng T., Xia W., Xu S. (2022). Prenatal and early postnatal exposure to ambient particulate matter and early childhood neurodevelopment: A birth cohort study. Environ. Res..

[25] Rivas I., Basagaña X., Cirach M., López-Vicente M., Suades-González E., Garcia-Esteban R., Álvarez-Pedrerol M., Dadvand P., Sunyer J. (2019). Association between Early Life Exposure to Air Pollution and Working Memory and Attention. Environ. Health Perspect..

[26] Yorifuji T., Kashima S., Diez M.H., Kado Y., Sanada S., Doi H. (2017). Prenatal exposure to outdoor air pollution and child behavioral problems at school age in Japan. Environ. Int..

[27] (2026). Ambient Air Quality Standards.

[28] Suades-González E., Gascon M., Guxens M., Sunyer J. (2015). Air Pollution and Neuropsychological Development: A Review of the Latest Evidence. Endocrinology.

[29] Block M.L., Elder A., Auten R.L., Bilbo S.D., Chen H., Chen J.C., Cory-Slechta D.A., Costa D., Diaz-Sanchez D., Dorman D.C. (2012). The outdoor air pollution and brain health workshop. Neurotoxicology.

[30] Win-Shwe T.T., Kyi-Tha-Thu C., Fujitani Y., Tsukahara S., Hirano S. (2021). Perinatal Exposure to Diesel Exhaust-Origin Secondary Organic Aerosol Induces Autism-Like Behavior in Rats. Int. J. Mol. Sci..

[31] McCloskey D.P., Croll S.D., Scharfman H.E. (2005). Depression of synaptic transmission by vascular endothelial growth factor in adult rat hippocampus and evidence for increased efficacy after chronic seizures. J. Neurosci..

[32] Kyi-Tha-Thu C., Fujitani Y., Hirano S., Win-Shwe T.T. (2022). Early-Life Exposure to Traffic-Related Air Pollutants Induced Anxiety-like Behaviors in Rats via Neurotransmitters and Neurotrophic Factors. Int. J. Mol. Sci..

[33] Patten K.T., González E.A., Valenzuela A., Berg E., Wallis C., Garbow J.R., Silverman J.L., Bein K.J., Wexler A.S., Lein P.J. (2020). Effects of early life exposure to traffic-related air pollution on brain development in juvenile Sprague-Dawley rats. Transl. Psychiatry.

[34] Calderón-Garcidueñas L., Solt A.C., Henríquez-Roldán C., Torres-Jardón R., Nuse B., Herritt L., Villarreal-Calderón R., Osnaya N., Stone I., García R. (2008). Long-term air pollution exposure is associated with neuroinflammation, an altered innate immune response, disruption of the blood-brain barrier, ultrafine particulate deposition, and accumulation of amyloid beta-42 and alpha-synuclein in children and young adults. Toxicol. Pathol..

[35] van den Hooven E.H., de Kluizenaar Y., Pierik F.H., Hofman A., van Ratingen S.W., Zandveld P.Y., Lindemans J., Russcher H., Steegers E.A., Miedema H.M. (2012). Chronic air pollution exposure during pregnancy and maternal and fetal C-reactive protein levels: The Generation R Study. Environ. Health Perspect..

[36] Nachman R.M., Mao G., Zhang X., Hong X., Chen Z., Soria C.S., He H., Wang G., Caruso D., Pearson C. (2016). Intrauterine Inflammation and Maternal Exposure to Ambient PM_2.5_ during Preconception and Specific Periods of Pregnancy: The Boston Birth Cohort. Environ. Health Perspect..

[37] Lee M., Shi L., Zanobetti A., Schwartz J.D. (2016). Study on the association between ambient temperature and mortality using spatially resolved exposure data. Environ. Res..

[38] Midouhas E., Kokosi T., Flouri E. (2018). Outdoor and indoor air quality and cognitive ability in young children. Environ. Res..

[39] Powell P.A., Rencz F., Carlton J., Schieskow S., Peasgood T., Mulhern B., Finch A., Herdman M., Verstraete J. (2026). Measuring health-related quality of life in infants and toddlers: Conceptual challenges and proposed recommendations. Qual. Life Res..

