# Supplementary figures and images for: Early-Life Exposure to Ambient Air Quality and Infant Health-Related Quality of Life: A Longitudinal Multi-Center Cohort in China
Source: Toxics. 2026 Apr 26;14(5):371. doi: 10.3390/toxics14050371 (PMC13211452; doi:10.3390/toxics14050371)

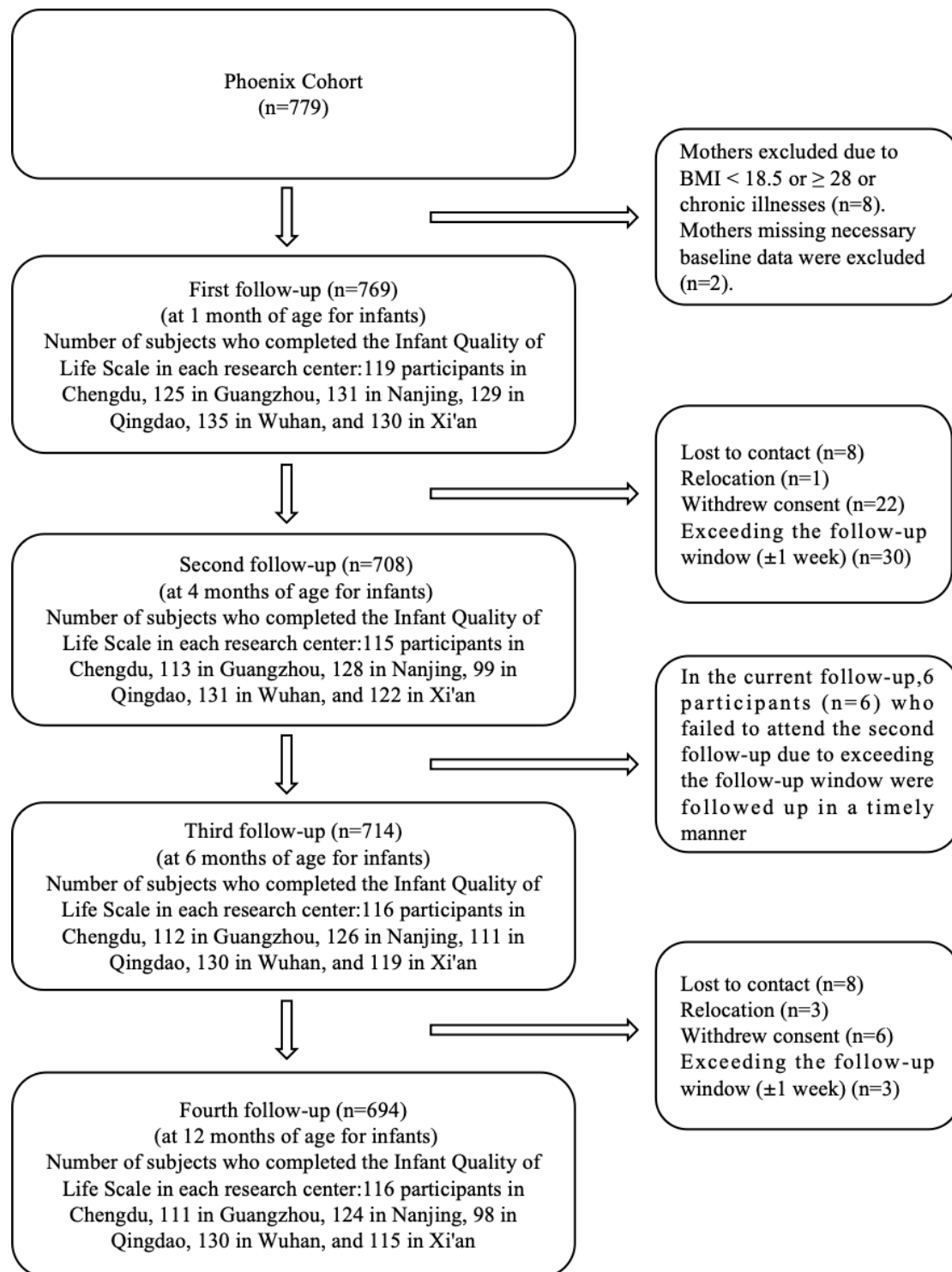

Figure S1. Screening and follow-up flow chart of study participants

Supplement: Supplementary file 1 [file toxics-14-00371-s001.zip › toxics-4247162-supplementary.pdf]
